# Supplementary figures and images for: From self-interest to collective action: The role of defaults in governing common resources
Source: PLoS One. 2025 Sep 11;20(9):e0331348. doi: 10.1371/journal.pone.0331348 (PMC12425191; doi:10.1371/journal.pone.0331348)

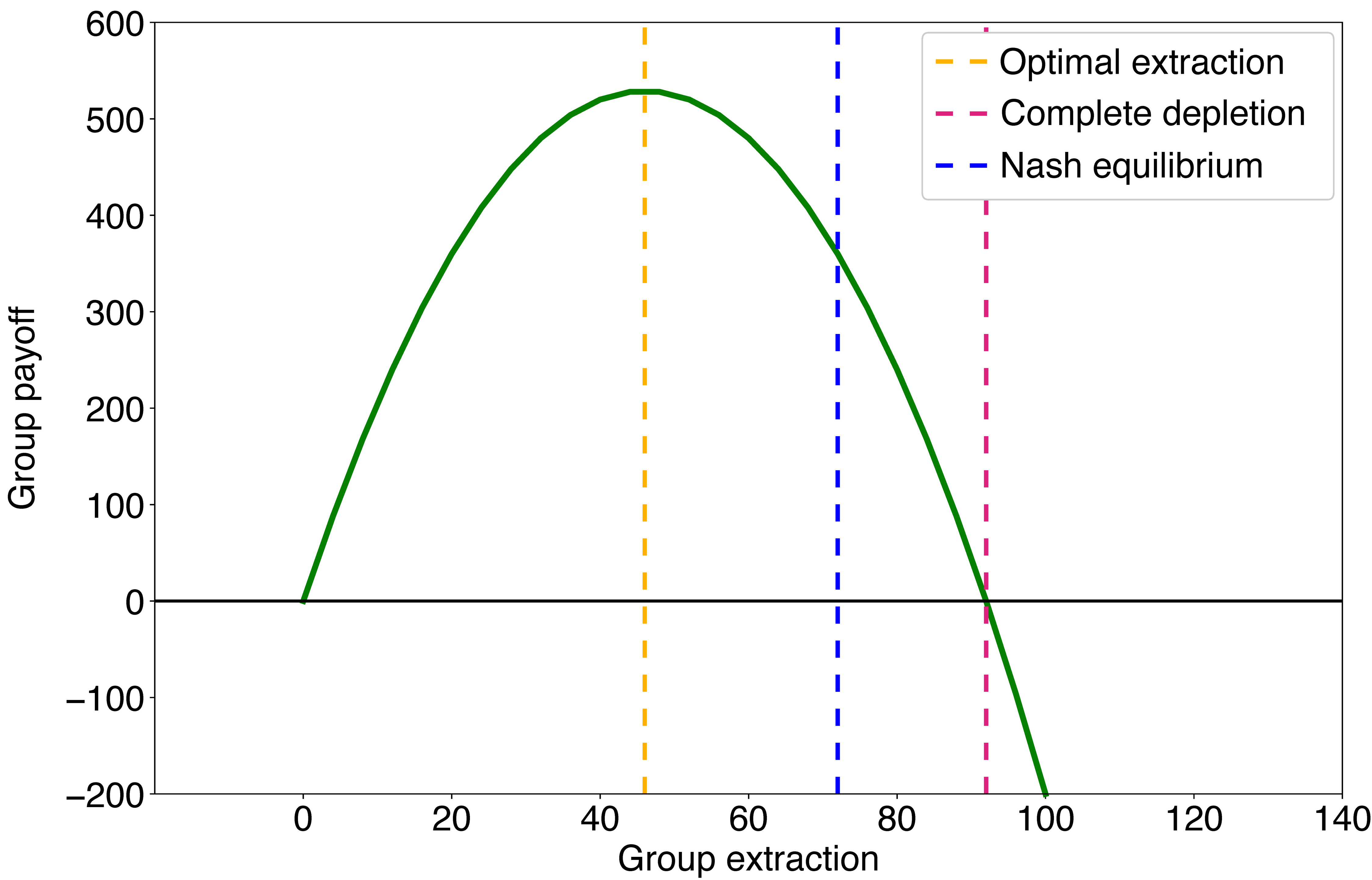

Supplement: S1 Fig — (TIF) [file pone.0331348.s001.tif]

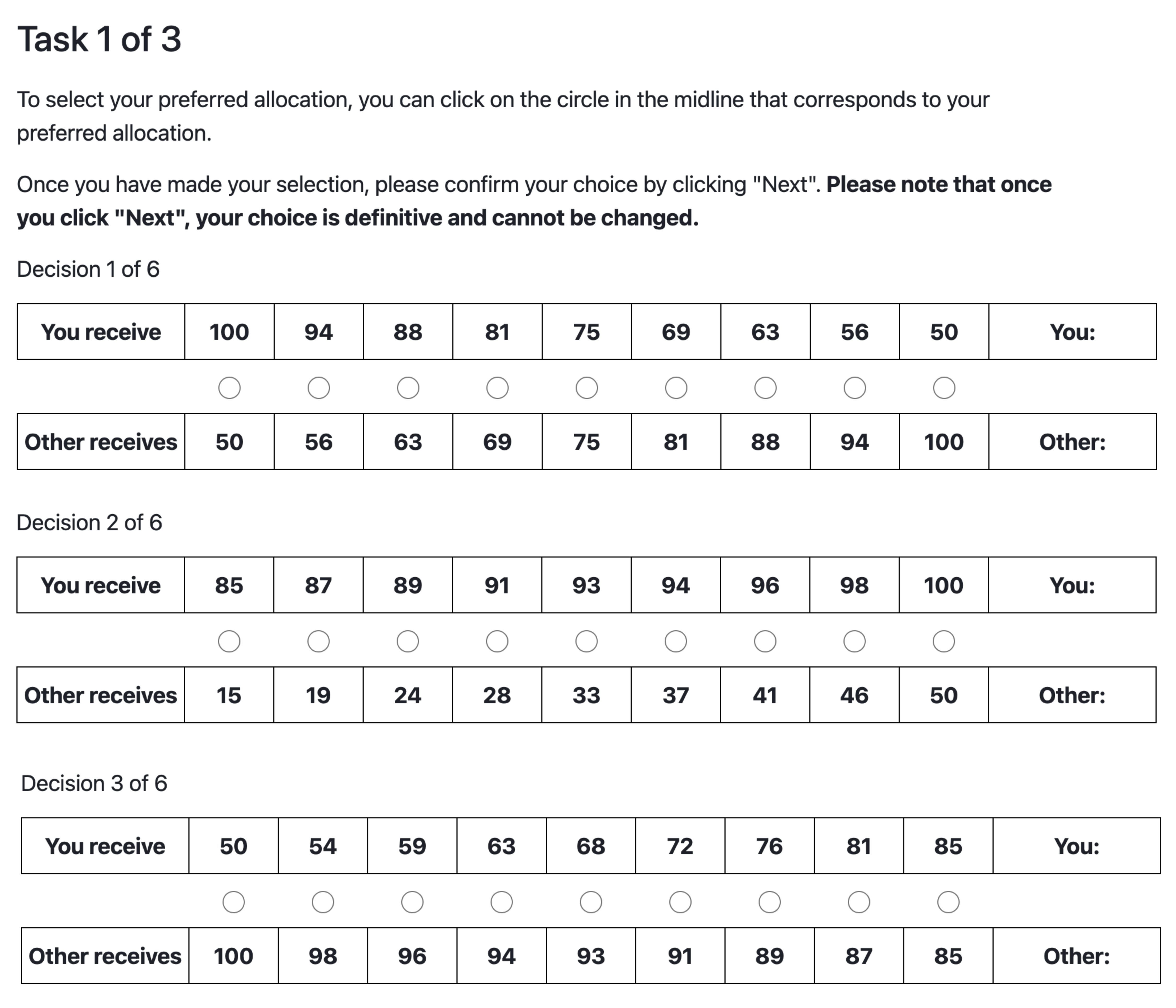

Supplement: S2 Fig — (TIF) [file pone.0331348.s002.tif]

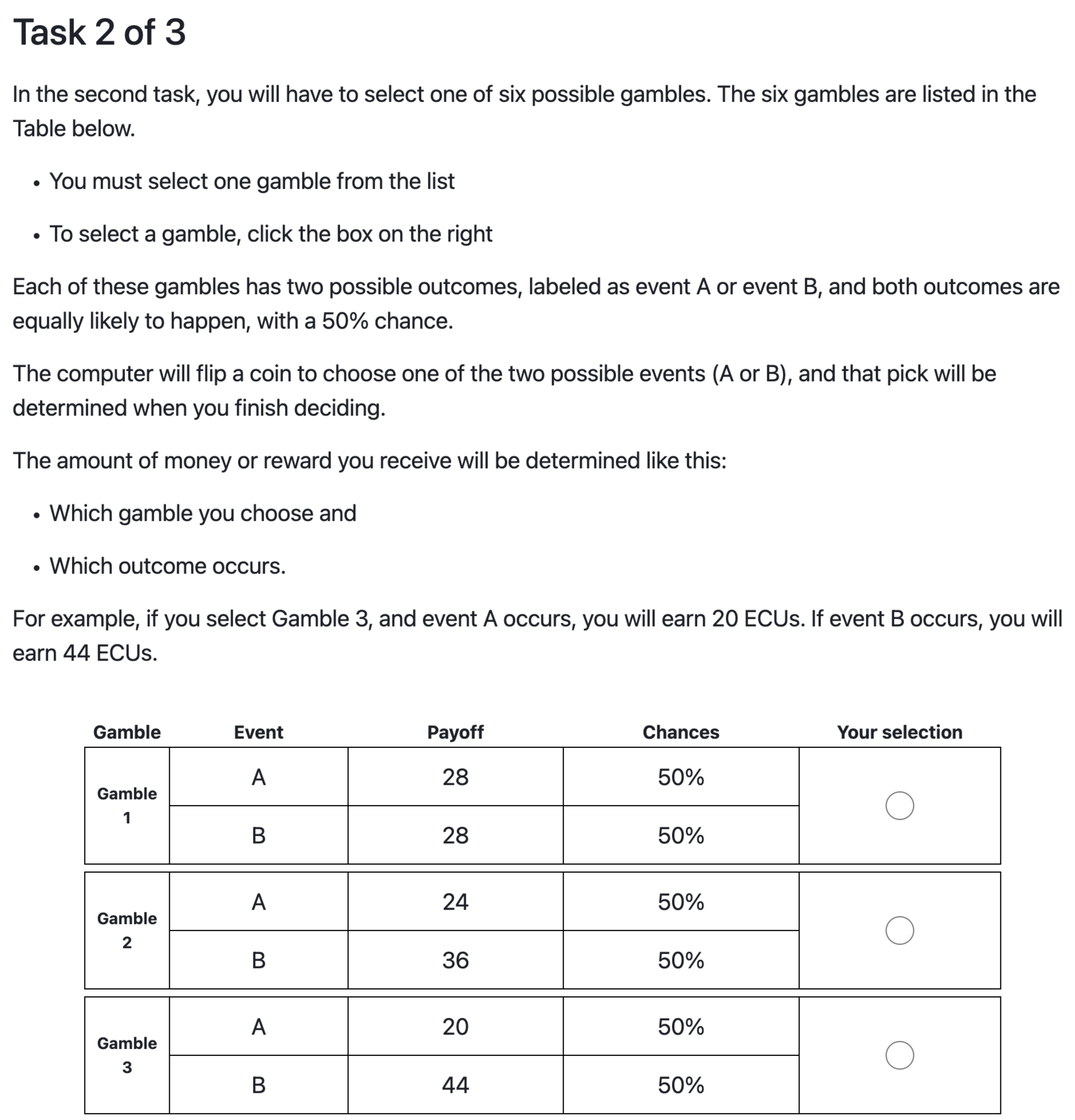

Supplement: S3 Fig — (TIF) [file pone.0331348.s003.tif]

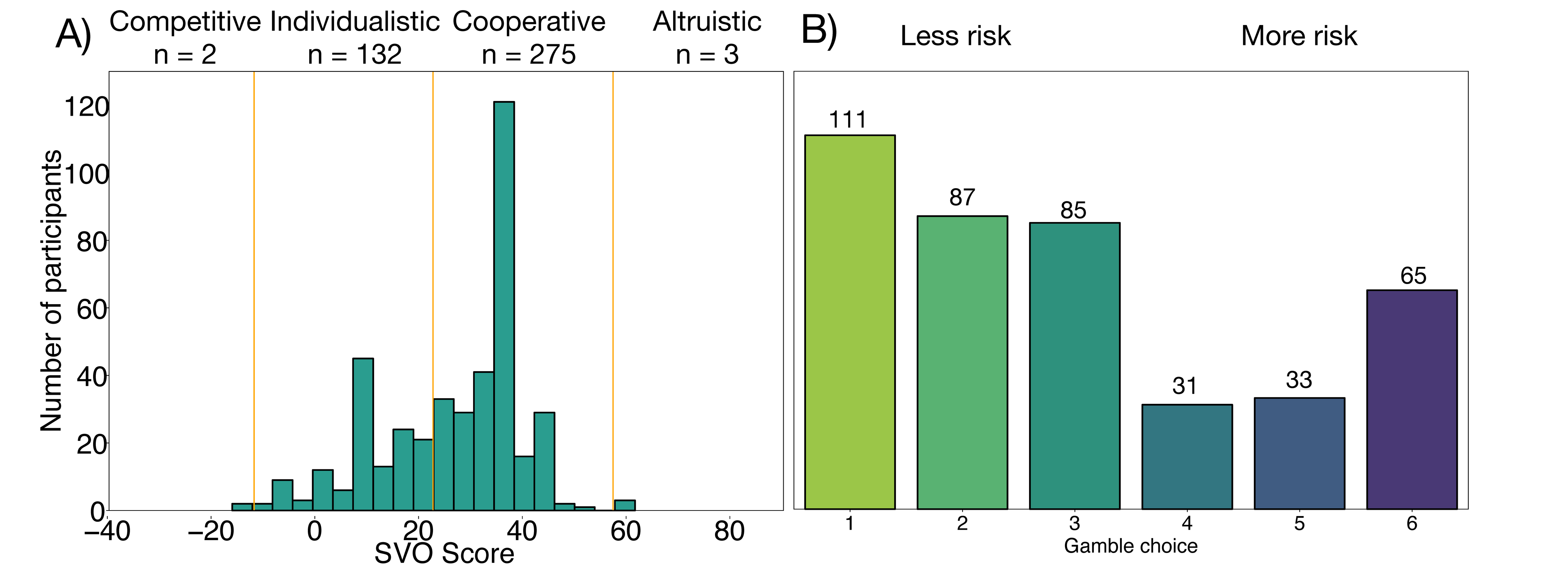

Supplement: S4 Fig — (TIF) [file pone.0331348.s004.tif]

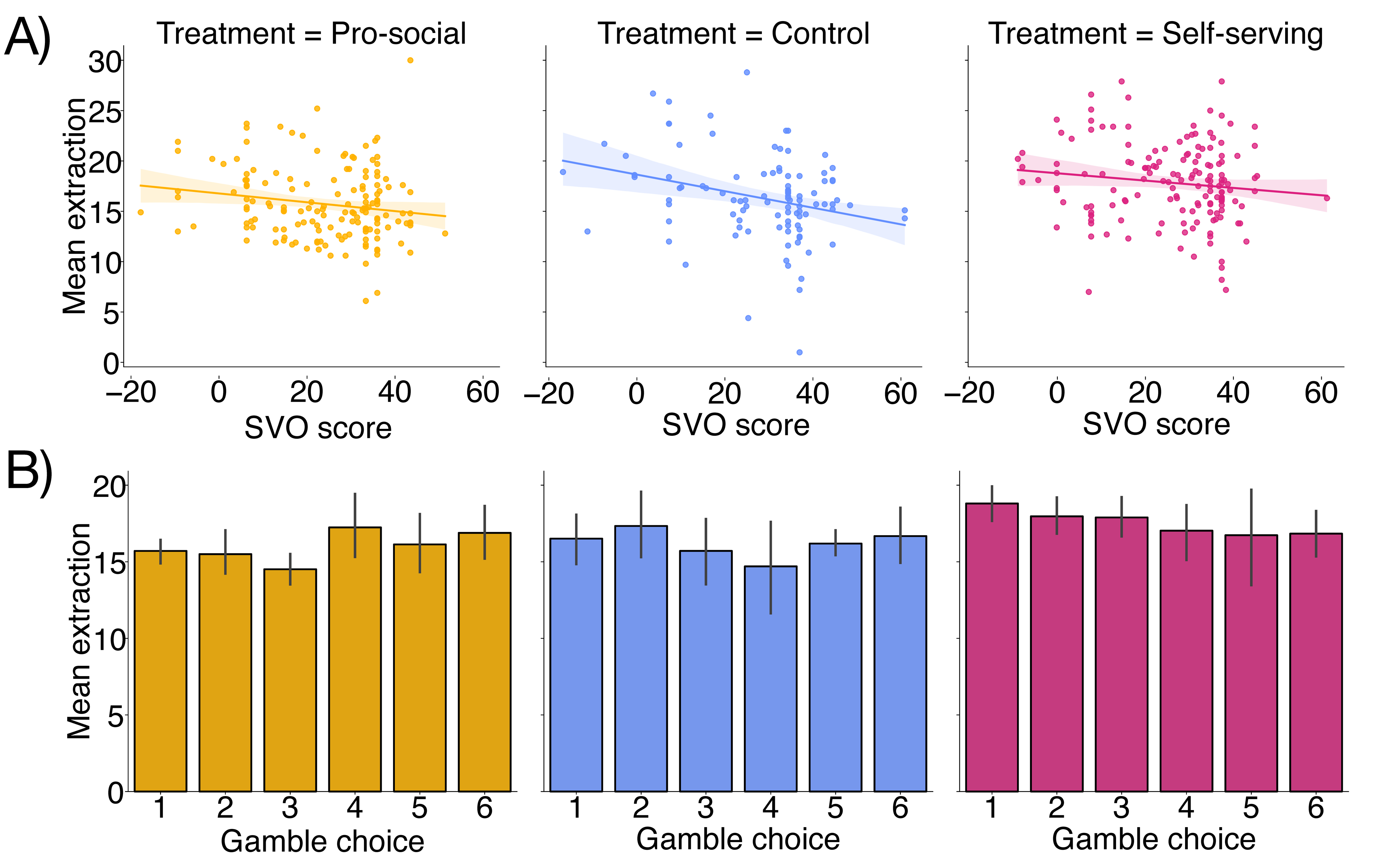

Supplement: S5 Fig — B: Mean extraction of the participants according to their gamble choices in Task 2 of the experiment. (TIF) [file pone.0331348.s005.tif]

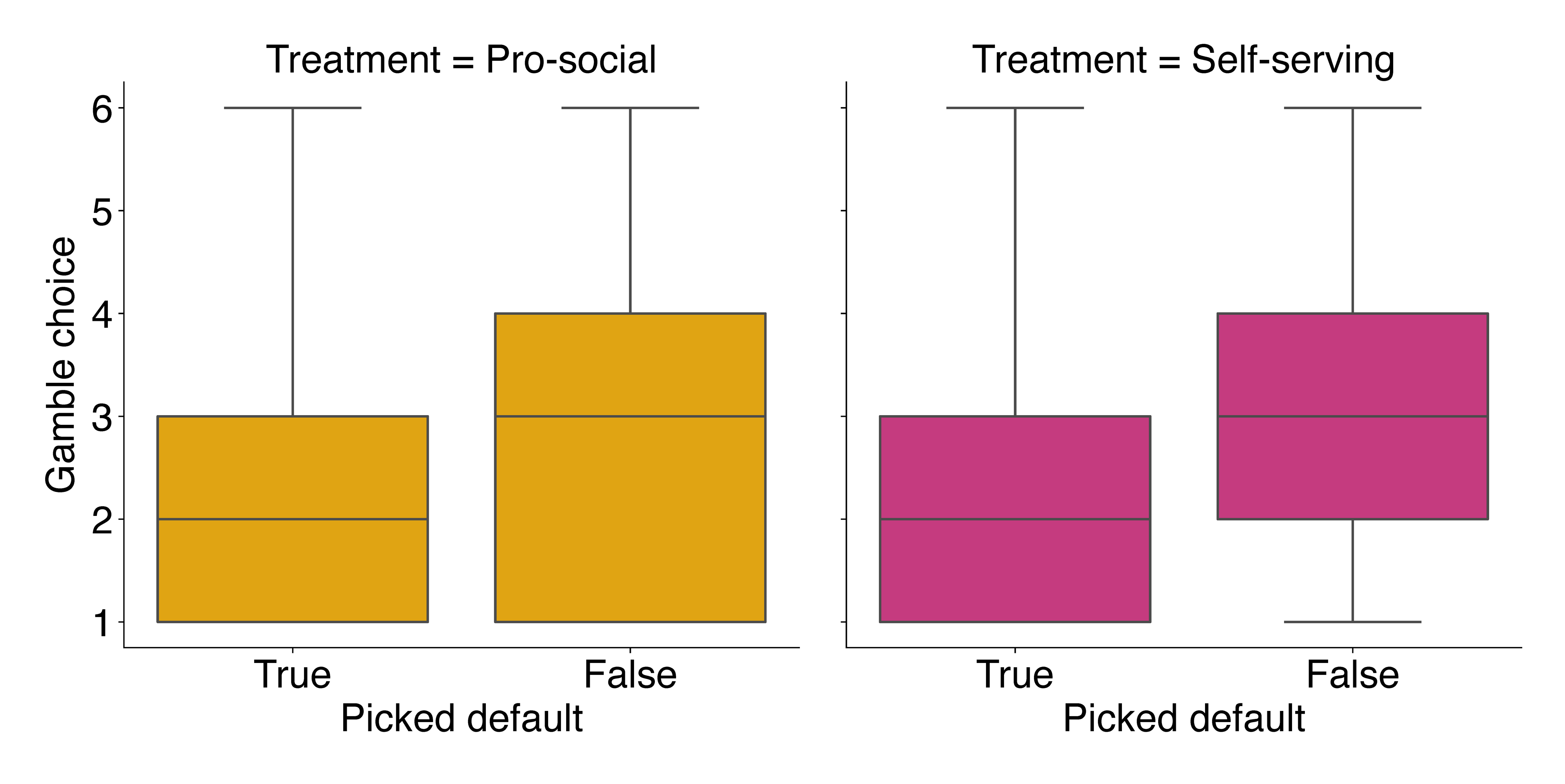

Supplement: S6 Fig — (TIF) [file pone.0331348.s006.tif]

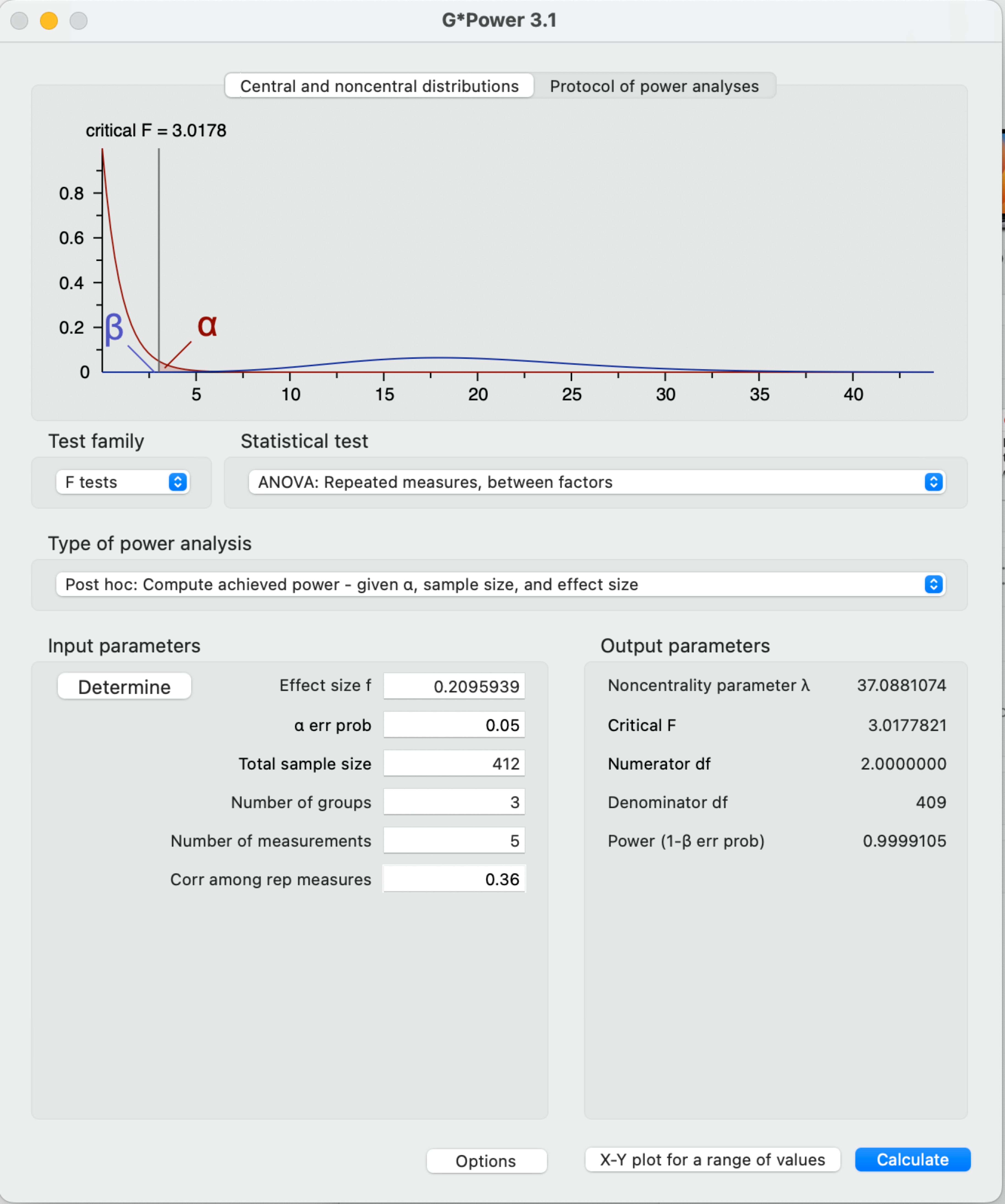

Supplement: S7 Fig — (TIF) [file pone.0331348.s007.tif]

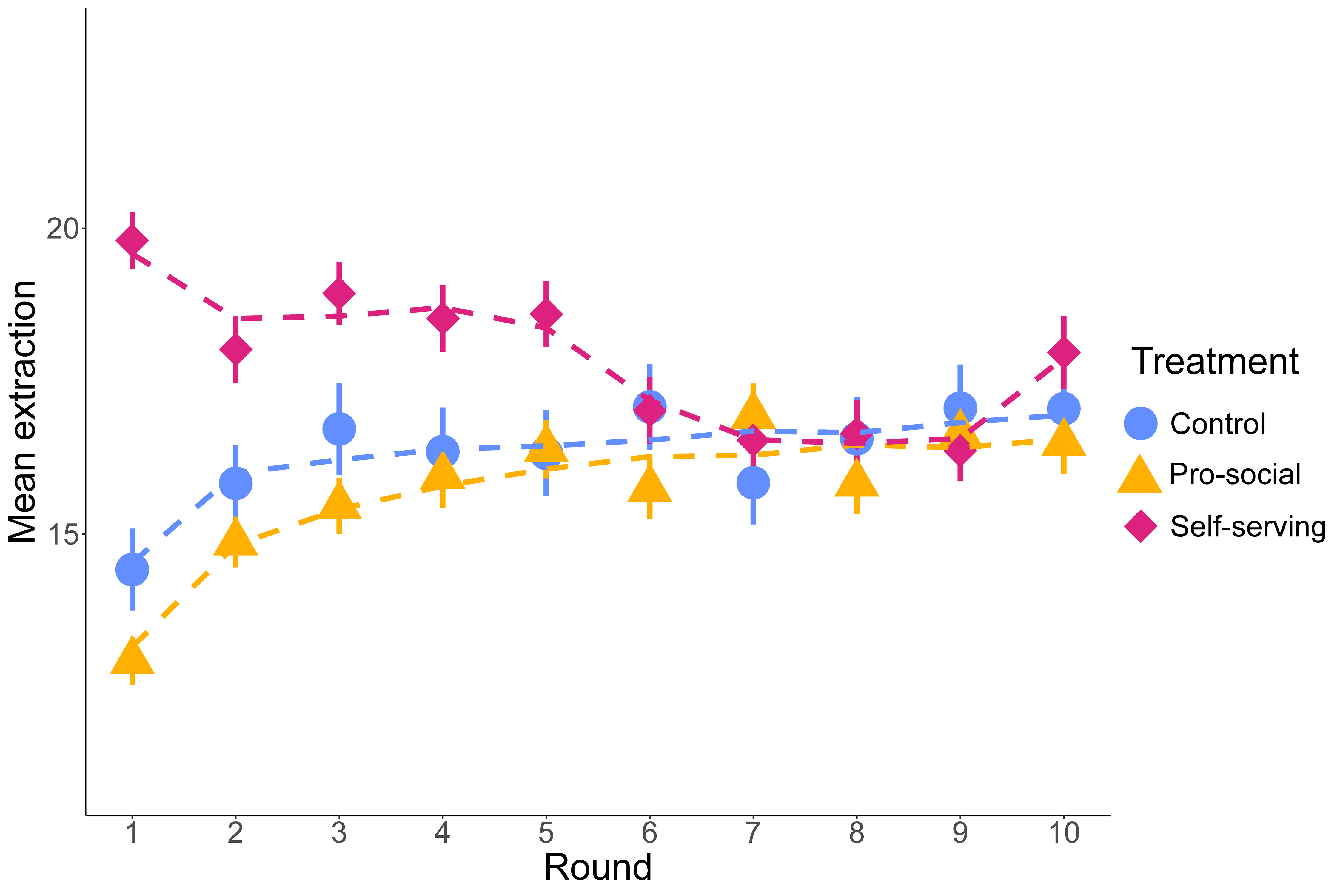

Supplement: S8 Fig — (TIF) [file pone.0331348.s008.tif]

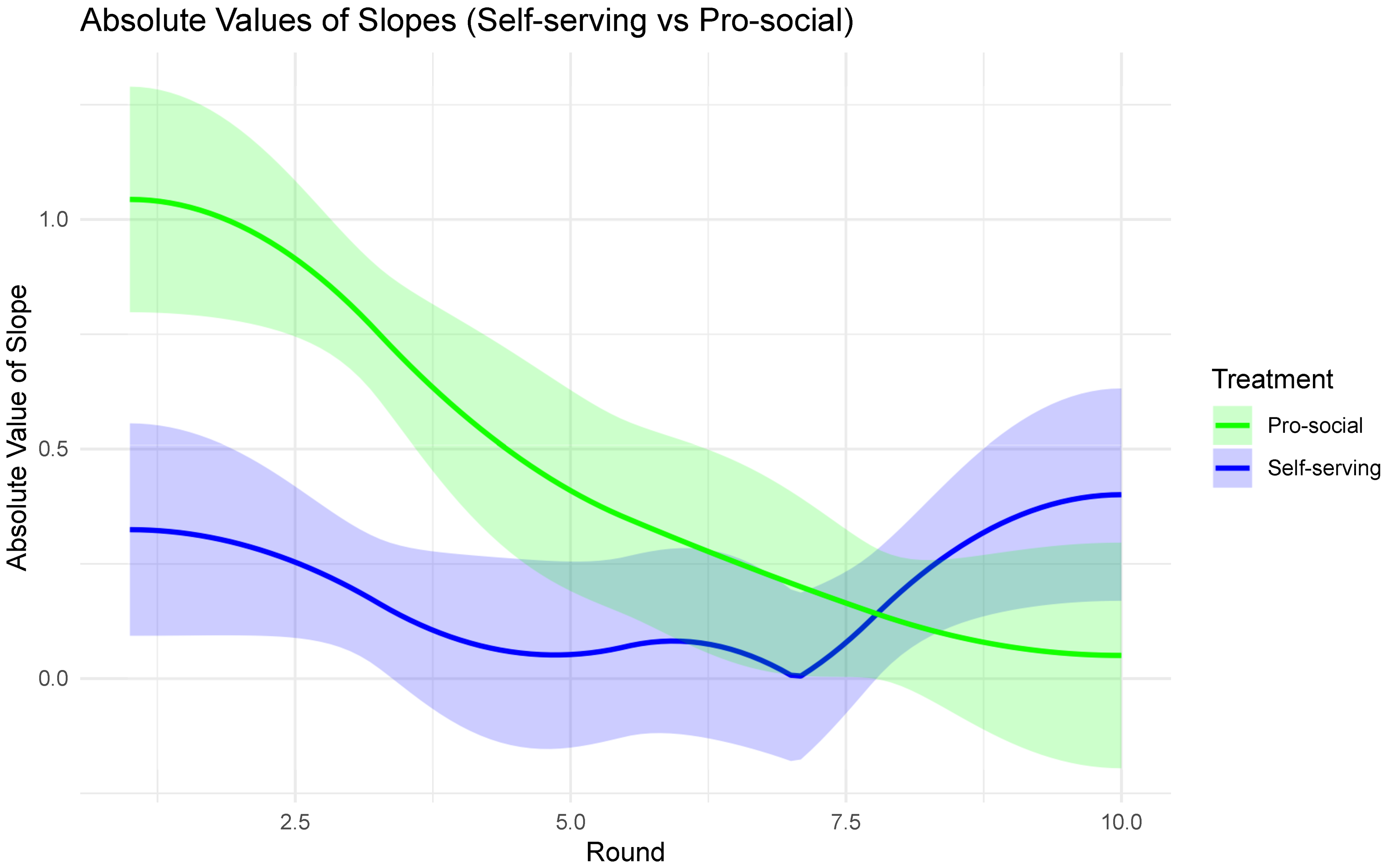

Supplement: S9 Fig — (TIF) [file pone.0331348.s009.tif]
