# Supplementary material for: From self-interest to collective action: The role of defaults in governing common resources
Source: PLoS One. 2025 Sep 11;20(9):e0331348. doi: 10.1371/journal.pone.0331348 (PMC12425191; doi:10.1371/journal.pone.0331348)
Supplement: Attachment — Submitted filename: Response to reviewers.pdf [file pone.0331348.s010.pdf]

Response from the authors

We thank the reviewers and the editor for the comments and concerns raised, this will make the results more robust and the paper more clear, we appreciate the detailed analysis and interest in our work. We responded to every comment made by the reviewers in this document in **blue letters** and the changes reflected in **red in the manuscript**.

Reviewer #1:

In this paper, the authors tested the effects of prosocial and self-serving defaults on extraction levels in a common-pool resource dilemma. The results of an online economic game showed that prosocial defaults caused people to extract less from the common pool and self-serving defaults caused people to extract more. However, these effects were short-lived, disappearing entirely when defaults were removed halfway through the game. The effects were also moderated by Social Value Orientation and risk preferences: for example, the authors found that prosocial defaults were more effective for individualists and risk-averse participants.

The study was described very clearly and the results were easy to follow. I really liked the statistical approach taken in the paper – more studies with repeated games should use GAMs to track changes across rounds! I also assessed the pre-registration, data, and analysis code as part of my review. The pre-registration was comprehensive and clearly laid out the key hypotheses. I was able to reproduce the statistical results of the paper with the data and analysis code (after some changes to file paths in the R file).

We appreciate the thorough analysis and interest that the reviewer has in our work. Below, we respond to each comment in blue and provide the location in the manuscript where the changes can be found and highlighted in red letters.

That said, I had a few concerns about the paper, mainly related to the discussion of prior literature, the statistical analyses, and the treatment of dropouts.

First, I felt that the discussion of prior literature was scant in places, making it difficult to assess how much of a contribution this paper is making. For example, in the Introduction section, the authors could have discussed how previous psychological work has already studied how the effects of defaults vary depending on individual differences in anxiety and avoidance (Zucchelli et al. 2024), cognitive effort (Ortmann et al. 2023),

and mood and personality (Manganari et al. 2022). In the Discussion section, it might also be worth discussing how the results are in line with other studies in behavioural economics, particularly studies showing that the effects of other interventions in repeated games often decay over time (e.g., Chaudhuri & Paichayontvijit, 2017) and go away entirely when removed (e.g., Fehr & Gächter, 2000). Regarding generalising the results to real-world settings, the authors could discuss the use of green defaults in airline carbon offsetting (Berger et al. 2022) and speculate as to whether such interventions might be more effective for more individualist and risk-averse customers. I am aware that the literature on defaults is very large, and **this is not a review paper!** But I think some extra discussion in places could better couch the current paper and its findings in the existing literature.

We thank the reviewer for taking the time and finding relevant literature for our paper. Indeed, this is not a review paper, but the sources provided can enrich our understanding of how defaults work and also give a solid background before presenting the results. We included the suggested references in the introduction and discussion sections of the paper. Find the changes in lines 121-127, 143-145 of the introduction and 550-552 and 559-560 in the discussion section.

Second, I had a few concerns with the analysis approach. While I liked the use of GAMs, the authors should consider including random intercepts for groups in the models in addition to the random intercepts for participants. The nested nature of the experiment means that there will be dependencies within groups. This group-level variation may not be fully captured by the “previous group extraction” fixed effect in the model. Do the results hold when this group-level variation is accounted for with additional random intercepts?

We thank the reviewer for this relevant observation. We agree that the random intercepts for our regression could capture the group-level variation. For this, we ran the GAM again, this time with a random effect for each group. You can find the results of the GAM in Table S8. We found that the results still hold, as the new random effect for the group-level is not significant. We added the new results, as well, we updated the formula in the Methods to include the new random effect variable.

Parametric Coefficients	Estimate	Std. Error	t value	Pr(> |t|)
(Intercept)	7.766495	0.366189	21.209	< 2e-16
TreatmentPro-social	-0.795405	0.531896	-1.495	0.1349
TreatmentSelf-serving	1.146488	0.533024	2.151	0.0315 *
extraction_last_round	0.018590	0.014787	5.542	2.80e-08 ***
others_extraction_last_round	0.006487	0.007870	0.824	0.4102
svo_score	0.292718	0.013670	21.413	< 2e-16 ***
gamble_choice	-0.121555	0.116483	-1.044	0.2968
**Smooth Terms**	**edf**	**Ref.df**	**F**	**p-value**
s(round):TreatmentControl	1.000e+00	1.0000	1.979	0.15955
s(round):TreatmentPro-social	2.187e+00	2.7346	5.197	0.00236 **
s(round):TreatmentSelf-serving	6.786e+00	7.9347	9.828	2.6e-16 ***
s(svo_score):TreatmentControl	8.471e-01	0.8471	192.971	2.6e-16 ***
s(svo_score):TreatmentPro-social	8.470e-01	0.8471	216.116	2.6e-16 ***
s(svo_score):TreatmentSelf-serving	8.470e-01	0.8471	205.772	2.6e-16 ***
s(player_id)	3.334e+02	405.000	2.658	<2e-16 ***
s(group_id)	7.699e-05	100.0000	0.000	0.99915

Table S8. Results from the GAM regression accounting for random effects given by the group heterogeneity, in this case, the random effects coefficient is at the bottom row of the "Smooth Terms" section.

Third, there is the perennial issue of dropouts in online experiments. If I'm understanding correctly, the authors' pre-registered approach was to exclude any participants who dropped out and any groups containing dropouts. But this is problematic from a causal inference perspective, especially if dropouts are more common in one treatment group compared to others – if this is the case, it becomes difficult to know whether differences between experimental conditions are due to the treatment itself or due to differences in attentiveness and characteristics of participants who drop out. The authors could remedy this in two ways: (1) by analysing whether dropout rates vary systematically across treatment groups, and (2) by running an "intention-to-treat" analysis (McCoy, 2017) that fits the same models but retains observations from groups that contain dropouts. Since there are only a few dropouts, the results will likely not differ all that much, but this analysis would show that the main causal conclusions are unbiased.

We thank the reviewer for the remark. Dropouts in behavioural economics can be divisive. While we initially assessed the impact of dropouts in the first two tasks, we did not consider their effect in the third task. To test this, we performed three different tests:

1. We fitted a logistic regression to make sure that no individual factor (such as demographics and results of the previous two tasks) was predicting individual drop out. None of the variables we considered was significant, which confirms that drop out was not linked with individual characteristics. Find the results in Table S2 in the Supporting Information.
2. Addressing your suggestions, we conducted a chi-square test to assess whether dropout rates differ significantly across treatments, and found no significant differences. Find this also in lines 428-432.

3. Finally, we re-fitted the GAM under an intention-to-treat framework by including dropouts and the remaining members of their groups. The main findings remain consistent, although some coefficients differ between the full-sample model and the one restricted to completed participants. The results are reported in Table S9.

Finally, here are some minor additional points to consider:

• Some visual examples of “free-riding” and “cooperation” in the economic game might be helpful for readers, especially those outside of behavioural economics.

Thank you for this comment, we included some examples of water use and grazing in lines 32-36

• I am assuming that the treatment is at the group-level, such that all participants within a group experience the same treatment. If this is the case, it should be clearly stated in the Methods section.

You are right, thank you for pointing this out. We added a clarification in the Methods section, in lines 263-264.

• Figures 1b and 1c are slightly confusing because the splines go in the opposite direction to the points in Figure 1a. For example, in Figure 1b, the spline goes below the horizontal line, even though the self-serving points are above the control points in Figure 1a. This may confuse readers. A simple fix is to flip the y-axis to capture “Self-serving - Control” instead.

Thank you for this comment, we are assuming you mean Figures 3b and 3c. Indeed, we were torn between showing “Control-Self-serving”, or to show “Self-serving-Control”. We decided to change both Figures 3b and 3c to keep consistency of “Treatment vs Control”.

• Four decimal places are used throughout – this is distracting and probably not necessary. Two decimal places would suffice.

Thanks for this observation, we removed the extra decimal places throughout the document, with the exception of the p-values, that the minimum is set to be at p = 0.001.

In summary, this was a clear and well-described paper that will contribute to the literature on default effects. Please let me know if you have any questions about this review.

Review signed: Scott Claessens (scott.claessens@gmail.com)

References

Berger, S., Kilchenmann, A., Lenz, O., Ockenfels, A., Schlöder, F., & Wyss, A. M. (2022). Large but diminishing effects of climate action nudges under rising costs. Nature Human Behaviour, 6(10), 1381-1385.

Chaudhuri, A., & Paichayontvijit, T. (2017). On the long-run efficacy of punishments and recommendations in a laboratory public goods game. Scientific Reports, 7(1), 12286.

Fehr, E., & Gächter, S. (2000). Cooperation and punishment in public goods experiments. American Economic Review, 90(4), 980-994.

Manganari, E., Mourelatos, E., Michos, N., & Dimara, E. (2022). Harnessing the power of defaults now and forever? The effects of mood and personality. International Journal of Electronic Commerce, 26(4), 472-496.

McCoy, C. E. (2017). Understanding the intention-to-treat principle in randomized controlled trials. Western Journal of Emergency Medicine, 18(6), 1075.

Ortmann, A., Ryvkin, D., Wilkening, T., & Zhang, J. (2023). Defaults and cognitive effort. Journal of Economic Behavior & Organization, 212, 1-19.

Zucchelli, M. M., Gambetti, E., Giusberti, F., & Nori, R. (2024). Use of default option nudge and individual differences in everyday life decisions. Cognitive Processing, 25(1), 75-88.

Reviewer #2:

Summary:

The paper reports the results of an experiment conducted on Prolific testing the effects of a pro-social and a self-serving default value for extraction decisions in a Common Pool Resource Dilemma (CPRD) game compared to a control condition without any default. The default was implemented simply as a value pre-selected in a drop-down menu of possible decisions, which participants could change easily if desired. The results indicate significant default effects compared to the control condition, with an interesting asymmetry: the self-serving default led to significantly higher extraction decisions in all five rounds of its implementation, whereas the pro-social default only led to lower extraction decisions in the first three rounds. Both types of defaults did not lead to any significant spillover effects after their removal (i.e., in rounds 6-10). Another interesting finding is that the pro-social default leads participants with individualistic or

competitive social preferences (as measured by the SVO measure) to extract less (whereas it has little effect on participants with cooperative and altruistic social preferences). Similarly, the self-serving default leads participants with cooperative or altruistic social preferences to extract more (with smaller effects on participants of individualistic or competitive type). Finally, the paper also finds that risk-averse participants are more strongly influenced by a default.

We thank the reviewer for the thorough feedback of our manuscript. Find the comments in blue after each point and the corrections in the manuscript can be found in red letters.

General Comments:
This is a well-written paper reporting the results of a well-designed and conducted experiment. The results are interesting, especially the analysis of heterogeneity in participants' reactions to defaults (as a function of social preferences and risk aversion). I am therefore in general quite positive about the paper; however, I do have some comments and questions that the authors should address in a revision.

Major Comments:
My major comments mainly refer to the reporting of the results. There is not always all relevant information provided to be able to understand and judge your analyses. Here are the specific points that I think should be addressed:

1. Did you use group averages in your analyses, or did you adjust for the non-independence of observations within the groups of four via clustering of standard errors? As participants interacted in groups of four and learned about each other's extraction decisions, the independent level of observation is the group of four (unless maybe in the very first round of the CPRD game). Please explain how you handled this, especially in your mixed effects model where you analyze the interactions between individual difference variables (SVO and risk preferences) and the experimental treatments.

We thank the reviewer for bringing this important point to our attention, which was also raised by the other reviewers. Indeed, we accounted for group interaction in our mixed effects model, but we included it in the Supporting Information, not in the main text nor in the mixed effects model formula. We added random intercepts for groups in addition to participants in the GAM. Given the nested design, we tested this adjustment and found the results unchanged, with the group-level random effect being non-significant. We changed this in the Methods section in lines 396-403 and added the table below (see Table S8 in the Supporting Information).

Parametric Coefficients	Estimate	Std. Error	t value	Pr(> |t|)
(Intercept)	7.766495	0.366189	21.209	< 2e-16
TreatmentPro-social	-0.795405	0.531896	-1.495	0.1349
TreatmentSelf-serving	1.146488	0.533024	2.151	0.0315 *
extraction_last_round	0.018590	0.014787	5.542	2.80e-08 ***
others_extraction_last_round	0.006487	0.007870	0.824	0.4102
svo_score	0.292718	0.013670	21.413	< 2e-16 ***
gamble_choice	-0.121555	0.116483	-1.044	0.2968
**Smooth Terms**	**edf**	**Ref.df**	**F**	**p-value**
s(round):TreatmentControl	1.000e+00	1.0000	1.979	0.15955
s(round):TreatmentPro-social	2.187e+00	2.7346	5.197	0.00236 **
s(round):TreatmentSelf-serving	6.786e+00	7.9347	9.828	2.6e-16 ***
s(svo_score):TreatmentControl	8.471e-01	0.8471	192.971	2.6e-16 ***
s(svo_score):TreatmentPro-social	8.470e-01	0.8471	216.116	2.6e-16 ***
s(svo_score):TreatmentSelf-serving	8.470e-01	0.8471	205.772	2.6e-16 ***
s(player_id)	3.334e+02	405.000	2.658	<2e-16 ***
s(group_id)	7.699e-05	100.0000	0.000	0.99915

Table S8. Results from the GAM regression accounting for random effects given by the group heterogeneity, in this case, the random effects coefficient is at the bottom row of the "Smooth Terms" section.

2. What do the p-values in square brackets ([…]) refer to? Is this the cluster-robust p-value? Or is this the p-value adjusted for multiple hypotheses testing? Please explain clearly how these p-values were derived.

We thank the reviewer for pointing this out. Indeed, initially, we included the p-values in brackets to account for the multiple hypotheses testing. We stated five different hypotheses in our pre-registration, and the p-values in brackets represented this adjustment. However, as we stated in the next question, we decided to deviate from this correction for multiple hypotheses (see the answer of question 3). We stated this more clearly in the Supporting Information section, lines 935-942 for clarification, and we removed the squared brackets all over the document.

3. In your pre-registration, you said that you would "account for multiple hypothesis testing by doing a correction on the tests such as Bonferroni correction". You do not seem to have done that in your analyses (at least it is not mentioned in the paper). Please explain whether (and how exactly) you adjusted for multiple hypotheses testing, or, if not, why you chose not to.

Thanks again for bringing up this important point. While our pre-registration mentioned a multiple testing correction, we did not apply one, as our hypotheses were pre-specified and limited in number. Following (Armstrong, 2014), such corrections are primarily warranted in exploratory analyses with many unplanned tests; in confirmatory settings, they risk inflating Type II errors. Since we defined the hypotheses and corresponding

tests in advance, rather than exploring the data post hoc or conducting a large number of unplanned comparisons, we considered the risk of inflated Type I error to be limited. Nevertheless, we acknowledge the deviation from the pre-registration and have now noted this in the manuscript in lines 935-942.

Minor Comments:

4. P. 2 you write: “For instance, an electricity provider can offer renewable energy for consumers by default, promoting a course of action supported by policy or a prevailing social norm. Yet, some consumers may prefer other sources of energy and adapt their consumption habits to accommodate their individual preferences [16, 17]. People who are particularly insensitive to the collective issue of energy over-consumption may be more likely to override a default…”. This makes it sound like the possibility to override defaults that do not correspond with personal preferences is a negative outcome. From the standpoint of libertarian paternalism (e.g., Thaler & Sunstein, 2003), however, this is one of the great things about defaults: people with preferences different from the choice architect’s objective are not forced to just succumb to the default, but can opt out according to their own preferences. Indeed, one of the undesirable side-effects of green defaults may be that not enough people opt out when their personal preferences differ (see Ghesla et al., 2020; I believe this is also the paper that should be cited as current reference 38).

Thank you for pointing this out, it is an important point. A mismatch between personal preferences and defaults does not necessarily lead to negative outcomes. Defaults offer a path that users may or may not follow, and this can benefit or harm collective outcomes. Our aim was to highlight that defaults are one of many strategies to promote renewable energy uptake. We revised this in the introduction (lines 69-71).

5. While I am myself positive about the potential of nudges or choice architecture interventions for behavioral change, when citing the paper by Mertens et al. (2022) on the effectiveness of nudges, I think it’s important to also cite the paper by Maier et al. (2022, PNAS) who, in a direct response to Mertens et al., argue that there is no meta-analytical evidence for the effectiveness of nudging after adjusting for publication bias.

We thank the reviewer for suggesting this paper. Indeed, one of the big issues in meta-analyses is the consideration of publication bias. We added this reference in line 89-90.

6. You provide a screenshot of the CPRD decision screen in Figure 2, but despite mentioning it in line 210, I couldn’t find further instructions or screenshots for the CPRD

in the supplementary material. Please provide these and ensure their location is clearly referenced.

We thank the reviewer for pointing out this mistake from our part. It seems that the Supporting information (that includes the instructions of the experiment) was not correctly included in the submission to PLOS One. Find the screenshots and the complete instructions now attached in Section S.08 in the Supporting Information.

7. I don't understand the relevance of the following sentence in the context of why the authors elicited participants' SVO and risk aversion: Line 131: "Moreover, certain decisions can pose greater challenges for specific individuals, such as the relatively slower evidence collection among equality seekers compared to categorical decision-makers [50]." Please elaborate or adapt this sentence for clarity.

Thank you for the comment. This is a publication from our team, where we use Drift Diffusion Models to show that reciprocal players (e.g., Tit-for-Tat) in Prisoner's Dilemma experiments deliberate longer—collecting evidence more slowly—than players using categorical strategies (e.g., All-C, All-D). We will add more context to clarify this point (see lines 182-184).

8. Why did you measure SVO and risk attitude before the CPRD experiment? In principle, these measures could serve as primes and lead to undesirable spillovers into the CPRD game. You can argue that this is no major problem, because Tasks 1 and 2 were constant across conditions; however, one might also argue that such priming could interact with the treatments (e.g., if measuring SVO primes people to be more cooperative, this could lead them to react more positively to the pro-social default). Personally, I do not think it's a major issue in the current experiment. However, in future work you might consider measuring such presumably stable individual preferences after the main experiment.

Thank you for raising this point. We deliberately chose to measure SVO and risk attitude before the CPRD task to avoid contamination from the experimental manipulation. Measuring the results of these two tasks after the main task could introduce bias, as participants' responses may be influenced by their experiences during the CPRD game, especially given its social and strategic nature. In contrast, collecting these measures beforehand ensures that they reflect baseline individual differences, not shaped by treatment effects. Moreover, while we recognise the theoretical concern about priming, we believe any such effects would be minimal and not significantly different across conditions, since the order of tasks was held constant. For this reason, any potential spillover would not confound the treatment comparisons central to our design. Please see changes in the Methods section, lines 370-378.

9. What is meant by “in the first rounds” in line 371? I assume you mean the first five rounds? If yes, please make this explicit. It is not completely clear, as you report an analysis for only rounds 3 to 5 just below on line 377.

This is true, thank you for suggesting this correction. We added the correction in line 441.

10. Some typos or suggested language edits:
a. Please proof-read the paragraph on study design (starting line 148) as there are several minor mistakes or language issues.
b. Line 205: I would suggest to formulate this sentence less strongly: “mitigating selective attrition concerns” (instead of “discarding”).
c. Line 489: “a strategic incentive” (not “an strategic incentive”)
d. Line 530: “cost-efffective tool” (not “cost-effect tool”)
e. Line 530: “to ease” seems to be a strange choice of words. Please consider rephrasing this.

We thank the reviewer for pointing out these typos, we edited the manuscript accordingly.

Congratulations to the authors on a nice paper and all the best for their future work!

References:
Ghesla, C., Grieder, M., & Schubert, R. (2020). Nudging the poor and the rich – A field study on the distributional effects of green electricity defaults. Energy Economics, 86, 104616.
Maier, M., Bartoš, F., Stanley, T. D., Shanks, D. R., Harris, A. J., & Wagenmakers, E. J. (2022). No evidence for nudging after adjusting for publication bias. Proceedings of the National Academy of Sciences, 119(31), e2200300119.
Thaler, R. H. and Sunstein, C. R. (2003). Libertarian paternalism. American Economic Review, 93(2):175-179.

Reviewer #3:

Review of “From self-interest to collective action: The role of defaults in governing common resources"

This paper studies the effect of positive and negative defaults on behavior in a common pool resource lab experiment. It further correlates these behavioral effects with social preferences measured in terms of social value orientation (SVO) and risk attitudes. The study finds that these defaults have corresponding effects on initial behavior, which then converge to the Nash equilibrium over time, and faster for the positive default than the negative default. No spillover effects were detected after defaults were lifted in the second half of the experiment. The study is interesting and can potentially add to the literature on default effects. My comments are as follows.

We thank the reviewer for this thorough read and feedback, find the responses to each point in blue and the amendments in the text in red.

Introduction/motivation: It is unclear how the renewable energy example on p.2 directly matched to the way defaults were experimentally implemented in terms of extraction values. There needs to be a better alignment of what the experiment does with what the paper claims it can tell us about which real-world applications. Clarifying the study's domain of defaults that it concerns is important, considering that the framing and potentially behavior vary also with the specific defaults studied.

We thank the reviewer for this comment, we assume you are referring to the example of the electricity provider in lines 65-69. Indeed, matching the results of behavioural experiments to real world applications have to be carefully done. We mentioned the domain of electricity as this is a very common application of green defaults, where readers can relate to the issue, but maybe it is better to rewrite this example to be more general, so there is no confusion. Find the correction in lines 69-74.

Also, how does this study contribute to resolving the mixed results mentioned in p.3? I was puzzled by how this "work expands on this current understanding by testing the presence and nature of spillover effects when default values are lifted." It was only later that I learned that the defaults were "lifted" in round 6 of 10 in all treatments, and this being a central feature of the experiment requires more emphasis and motivation in the introduction. Generally, I think the first section needs a careful rewrite to elucidate the contribution of the paper.

Thank you for the comment. It's important that readers understand the experiment and key hypotheses—such as spillover effects of defaults—before the results. Our study contributes to this debate, finding no post-treatment effects, in line with Cappelletti et al. (2014) but contrasting Ghesla et al. (2019). As requested, we revised this section (see lines 108-110).

2) Experiment: While I understand the practicalities of excluding data from dropouts in the sample, and selecting those with high approval ratings, I wonder if this leads to a selection bias whereby certain types are self-excluded from the tests. It would be good to explain if this imposes any limitations on the findings and to explicitly defend the integrity of the experiment in this respect, if possible.

We thank the reviewer for raising these points, which was also noted by Reviewer 1. First, we restricted recruitment to participants with high approval ratings to increase the chances that they would complete the experiment. We are aware that this might impose a selection bias on the results, but it was a necessary practical step to ensure data quality and reduce the risk of incomplete responses. To make sure there is no confounding factor affecting the results, we tried three different approaches:

1. We fitted a logistic regression to make sure that no individual factor (such as demographic information and results of the previous two tasks) was contributing for the participants to dropout of the experiment in the third task. We found no evidence of this. Find the results in Table S2 in the Supporting Information.
2. Additionally, we conducted a chi-square test to assess whether dropout rates differ significantly across treatments, and found no significant differences. Find this also in lines 428-432.
3. Finally, we re-fitted the GAM under an intention-to-treat framework by including dropouts and the remaining members of their groups. The main findings remain consistent, although some coefficients differ between the full-sample model and the one restricted to completed participants. The results are reported in Table S9.

Note that this analysis was a suggestion from reviewer 1.

3) Analysis: I was unable to tell if the non-independence from repeated interaction was considered in the reported tests. For example, how was an independent observation defined in the non-parametric tests, and were clusters used in the multivariate tests?

Thank you for bringing this up. We accounted for non-independence of repeated interactions in two ways: first, in the power analysis, we assumed low correlation between repeated measures (see Supporting Information and Pre-registration for more details). Also, in our mixed-effects model, non-independence of repeated interactions is addressed through the inclusion of random effects at both player (which account for the repeated measures of the same individual) and groups level.

Also, the tests for behavior in the first and second halves of the experiment should be analyzed separately and the same applies for the presentation of their results and implications.

We analysed the data separately, which is why we report findings for the first five rounds in section 4.1—focusing on average extractions per treatment—and the second half in section 4.2, to show changes once the default is lifted. Analysing the full game in the mixed-effects model was useful to capture the difference between the treatment and the control. For example, it highlighted that in the Pro-social treatment, extending the default had no effect compared to the control.

On the matter of SVO types, are the distributions comparable with those of previous studies (related to my previous point 2) and across treatments? There were only three altruistic subjects so I would be extremely cautious about interpreting their “results”, while the present analysis (text and figures) treats them as prominently as dominant groups of individualists and cooperators. Section 4.3 mentions a hypothesis that was not previously developed.

Thank you for this comment. The aim of our study was not to analyse SVO types in detail, but to explore whether personal preferences relate to extraction behaviour. We agree that caution is needed with small subsamples like altruists, which is why we grouped them with other pro-socials as "cooperatives". A similar approach was used in a similar experiment by (Guido et al., 2023), where they grouped SVO’s angles as two categories, “Individualistic” = 36.9% and “Prosocial” = 63.1%. In our sample, we have 67.7% of Cooperatives and 32.3% Individualistic. A balanced comparison across SVO types would require a larger, targeted sample (for example (Brucks & Van Lange, 2007)), which falls outside the scope of this study.

4) The self-serving default has a noticeable negative effect on cooperation and convergence to the Nash equilibrium is slow, thus reflecting the dangers of such a default. This is also the case in the control, albeit to a lesser extent. In contrast, the pro-social default has the least negative effect on cooperation. This practical implication deserves more emphasis in the discussion.

We appreciate this comment, we added more emphasis in the Discussion section in lines 561-562.

Minor comments:
In the composite figures 1 and 3, the inconsistency of labels for A-D in caption and a-d in figure is a little confusing.

Thank you, we have corrected the captions and now it reflects the correct labels in the figures.

References

Armstrong, R. A. (2014). When to use the Bonferroni correction. *Ophthalmic and Physiological Optics*, *34*(5), 502–508. https://doi.org/10.1111/opo.12131

Brucks, W. M., & Van Lange, P. A. M. (2007). When Prosocials Act Like Proselfs in a Commons Dilemma. *Personality and Social Psychology Bulletin*, *33*(5), 750–758. https://doi.org/10.1177/0146167206298569

Guido, A., Tverskoi, D., Gavrilets, S., Sánchez, A., & Andrighetto, G. (2023). *Nudging or Nagging: The Perils of Persuasion*. https://doi.org/10.2139/ssrn.4404960
