# Supplementary material for: From self-interest to collective action: The role of defaults in governing common resources
Source: PLoS One. 2025 Sep 11;20(9):e0331348. doi: 10.1371/journal.pone.0331348 (PMC12425191; doi:10.1371/journal.pone.0331348)
Supplement: Attachment — Submitted filename: Response to reviewers PLOS One 2.pdf [file pone.0331348.s011.pdf]

Response to reviewers

We thank the reviewers and the editor for the comments and concerns raised, this will make the results more robust and the paper more clear, we appreciate the detailed analysis and interest in our work. We responded to every comment made by the reviewers in this document in **blue letters** and the changes reflected in **red in the manuscript**.

Reviewer #1:

Thank you to the authors for dealing with all of the points that I raised in my previous review. I am happy to recommend this paper for publication at PLOS One. I hope that it will make a positive contribution to the literature on default effects.

There is one remaining minor issue that should be addressed before publication. It looks like the logistic regression testing the associations between demographics and drop out rates (reported in Table S2) may not have converged properly, since the standard errors for many of the parameters are huge. This may be because there are only a small number of dropouts in the dataset. The authors should check whether the results of this model are valid.

We thank the reviewer for carefully checking our previous edits and responses. Regarding Table S2, we agree that several variables (such as nationality and age) exhibit large standard errors. This is primarily due to (1) the small number of dropouts (21 out of 709 participants) and (2) the limited contribution of these variables to the binomial regression. This supports our argument that neither demographic factors nor prior task performance significantly influenced participant dropout.

To further examine this, we fitted a reduced logit regression including only task-related variables (SVO score and gamble choice). We found no significant association between SVO score or gamble choice and dropout (see Table S3). We thank the reviewer for prompting this analysis, which led to an additional insight into participant behaviour on Prolific. Find the changes in lines 258-261.

Reviewer #2:

I would like to thank the authors for the revision, the changes they made to the paper, and their responses to my first-round comments. Most of my comments have been addressed. I have the following remaining comments relating to points already raised in the first review.

1. In your response letter, you explain that you've accounted for non-independence in your mixed-effects model by including random intercepts for groups, which is appropriate. As a robustness check, however, I encourage you to report cluster-robust standard errors at the group level to show that your key ppp-values remain unchanged even if the random effect is negligible.

We thank the reviewer for pointing this out, We used robust standard errors clustered at the group level in our GAM. When accounting for potential intra-group correlation, the initial parameters (e.g. Treatment, SVO score and gamble choice smoothed by round) remained significant (see Supplementary Table S11), confirming the robustness to non-independence of observations within a group. We mentioned this in the text in lines 521-526.

Additionally, your reply does not address how you handled non-independence in the non-parametric tests and ANOVAs reported in the Results section. Please specify on how many observations each of these tests is based, and describe what adjustments (e.g., aggregation to group means or clustering corrections) you applied to account for within-group dependence.

We thank the reviewer for this comment. We changed the statistical tests used (ANOVA's and KS tests) to account for the non-independence of the observations by computing group averages across rounds, and made our tests on these averages. We find that all statistical tests hold but one, where we state that extractions in the Self-serving treatment by Risk-seekers participants were higher than Risk-averse ones. Despite this, the main results of our paper hold, as shown with the GAM regressions.

Moreover, to make sure that those statistical tests are still robust, we fitted the relevant ANOVA's taking only the first round, where behaviour cannot yet be correlated within a group. We also find that the results still hold, but one, where we state that Risk-averse participants extracted less in the Pro-social treatment than Risk-seeking ones. Find this analysis in Section S0.8.

We added this new information about the analysis in Section S0.1.1 (Deviation from the pre-registration).

2. I see no major problems with your deviations from the pre-registration as outlined in S0.1.1 in the Supporting Information. However, I think you should at least put a reference to this section in the main body of the paper (e.g., on line 213, when mentioning the pre-registration).

Thanks for pointing this out. In our text, when we mention the OSF Preregistration we also point to the deviations section in our Supplementary Information. Find the changes in lines 213-215.

3. Reference #47 should be changed to the following: Ghesla, C., Grieder, M., & Schubert, R. (2020). Nudging the poor and the rich – A field study on the distributional effects of green electricity defaults. Energy Economics, 86, 104616.

Thank you for this, perhaps we pointed out to the wrong reference of Ghesla, the one in line 74 should be updated and it’s now the reference number 22.
